# *In Vivo* and *Ex Vivo* Pediatric Brain Tumor Models: An Overview

**DOI:** 10.3389/fonc.2021.620831

**Published:** 2021-04-01

**Authors:** Zhiqin Li, Sigrid A. Langhans

**Affiliations:** Nemours Biomedical Research, Alfred I. duPont Hospital for Children, Wilmington, DE, United States

**Keywords:** medulloblastoma, glioma, pediatrics, preclinical models, *in vivo* models, *in vitro* models, cancer

## Abstract

After leukemia, tumors of the brain and spine are the second most common form of cancer in children. Despite advances in treatment, brain tumors remain a leading cause of death in pediatric cancer patients and survivors often suffer from life-long consequences of side effects of therapy. The 5-year survival rates, however, vary widely by tumor type, ranging from over 90% in more benign tumors to as low as 20% in the most aggressive forms such as glioblastoma. Even within historically defined tumor types such as medulloblastoma, molecular analysis identified biologically heterogeneous subgroups each with different genetic alterations, age of onset and prognosis. Besides molecularly driven patient stratification to tailor disease risk to therapy intensity, such a diversity demonstrates the need for more precise and disease-relevant pediatric brain cancer models for research and drug development. Here we give an overview of currently available *in vitro* and *in vivo* pediatric brain tumor models and discuss the opportunities that new technologies such as 3D cultures and organoids that can bridge limitations posed by the simplicity of monolayer cultures and the complexity of *in vivo* models, bring to accommodate better precision in drug development for pediatric brain tumors.

## Introduction

Brain tumors are the most common solid tumors and the leading cause of cancer-related death in children. The incidence and mortality rate of primary brain and other central nervous system tumors have not changed significantly in recent years, with an average incidence rate of 5.65 per 100,000 population and an average mortality rate of 0.72 per 100,000 population for the 0 to 14 years age group from 2011 to 2015 in the United States (1). In the past, the diagnosis and classification of brain tumors had largely relied on histological characteristics derived from hematoxylin and eosin-staining, and immunohistochemical detection of lineage-associated proteins. However, more and more evidence shows that histologically similar brain tumors sometimes have distinct molecular features; they respond differently to the treatment and have various prognosis as well. In addition, some histologically ambiguous tumors may largely rely on their molecular characterization for their diagnosis and treatment plan. In 2016, the World Health Organization (WHO) updated classification of central nervous system tumors by incorporating molecular features into traditional histological characteristics for more accurate diagnosis, prognosis predictions, and treatments (2–6). With the overall success rate of new anticancer drugs remaining low (7, 8), we will need to switch from “one size fits all” treatments to more specific individualized strategies, to increase treatment efficacy, to reduce complications due to treatment, and to improve the translation rate of anti-cancer drugs. Brain tumor models that can mimic tumor initiation and progression, and predict a tumor’s response to treatments *in vivo* are fundamental to achieve this goal. In this review, we provide an overview of the most common pediatric brain tumors and currently available well-annotated *in vitro* and *in vivo* tumor models. We also discuss the advantages and limitations of each model, which need to be considered when choosing an appropriate tumor model that best suits the experimental purpose.

### Common Pediatric Brain Tumors and Molecular Subgrouping

#### Gliomas

Glioma is the most common pediatric primary brain tumor, representing approximately 47% of brain tumor cases in the age group of 0-19 years. Glioma can originate from all glia cell types and 75% of these glial tumors are astrocytoma (1). Glioma are highly heterogeneous tumors, ranging from low-grade glioma (LGG) to high-grade glioma (HGG) depending on the tumor malignant status.

LGG is the most common glioma, which is typically nonmalignant and slow growing. Histologically, LGGs include pilocytic astrocytoma (PA), pilomyxoid astrocytoma (PMA), oligoastrocytoma, subependymal giant cell astrocytoma (SEGA), pleomorphic xanthoastrocytomas (PXA), oligodendroglioma, ganglioglioma, dysembryoplastic neuroepithelial tumors, etc., among which pilocytic astrocytoma is the most common form. The aberrant Ras-mitogen activated protein kinase (MAPK) signaling pathway is mainly reported in LGG. The mutations usually occur at BRAF in this pathway, including the KIAA1549-BRAF fusion and BRAF V600E mutant, which lead to constitutive activation of the MAPK pathway. Furthermore, kRAS, FGFR1, MYB/MYBL1, NTRK2, NF1, TSC1/2 and other genetic alteration have also been identified in pediatric LGG. Unlike adult LGG, IDH mutations are almost absent in children (3–6, 9–11). In some cases, the molecular alteration is associated with a specific tumor type. For example, KIAA1549- BRAF fusion is mostly found in pilocytic astrocytoma (PA), while BRAF V600E is frequently detected in pleomorphic xanthoastrocytoma (PMA) and gangliogliomas (12).

HGG is relatively uncommon in pediatric glioma, accounting for around 20% of cases. However, HGGs are diffusely infiltrating malignant tumors and they are usually aggressive with an overall very poor prognosis; some patients succumb to the tumor within one year after diagnosis. Based on distinct histological and radiological features, HGG is subclassified into anaplastic astrocytoma, diffuse intrinsic pontine glioma (DIPG) and glioblastoma multiforme (GBM). Mutations in histone genes were first discovered in pediatric HGGs, and now serve as a hallmark of this glioma type. Histone mutations often vary according to HGG locations. In tumors arising from the midline and pons, K27M mutations in H3F3A (encoding histone H3.3) or HIST1H3B/C (encoding histone H3.1) are very common, which lead to a global decrease of H3 K27 trimethylation by inhibiting polycomb repressive complex 2 (PRC2) activity through sequestration of its catalytic subunit EZH2; while G34R (or rarely G34V) mutations in H3F3A (encoding histone H3.3) are mostly reported in hemispheric HGGs. In addition, the RTK/RAS/PI3K pathway (e.g., PDGFRA, PIK3CA, PIK3R1, or PTEN) and the p53/Rb pathway (e.g., TP53, CDKN2A, CDK4/6, CCND1-3) are also dysregulated in pediatric HGG (3, 4, 9–11, 13–15). Recent studies discovered some overlap in molecular profiling between LGG and HGG. BRAF V600E and FGFR1 mutations are found both in LGG and HGG (9, 10), which suggests that LGG and HGG might share a similar biological mechanism of tumor pathogenesis.

#### Ependymal Tumors

Ependymomas represent 5.5% of all pediatric primary brain tumor cases in the age group of 0 to 14 (1). Ependymomas are thought to originate from radial glia cells of the ependymal lining of the ventricles and the central canal. Histologically, ependymomas are classified into 4 groups: subependymoma, myxopapillary ependymoma, classic ependymoma, and anaplastic ependymoma, of which classic and anaplastic ependymoma are the most common subtypes in children. Classic ependymoma is further subclassified into 3 subtypes: papillary, clear cell, and tanycytic ependymoma based on their histological features (4, 5).

The molecular characteristics of ependymoma is usually associated with its location. Over 90% of pediatric ependymomas arise in the infratentorial and supratentorial regions. The infratentorial posterior fossa (PF) ependymomas are generally subclassified into Group A (PF-EPN-A) and Group B (PF-EPN-B) based on their DNA methylation profiling. PF-EPN-A tumors are hypermethylated, and mostly found in infants and young children, who have a poorer outcome compared to those with PF-EPN-B tumors, which are typically seen in adolescents and adults. Supratentorial (ST) ependymomas in children have two major subgroups: RELA fusion-positive (ST-EPN-RELA) ependymoma and YAP1 fusion-positive (ST-EPN-YAP1) ependymoma. ST-EPN-RELA usually harbors the fusion protein of C11orf95 and RELA, which constitutively activates the NF-κB pathway by enriching a RELA-encoded transcription factor p65. In ST-EPN-YAP1 ependymoma, transcriptional coactivator YAP1 fuses with other genes such as MAMLD1 and FAM118B and can upregulate Notch signaling. Compared to ST-EPN-YAP1, ST-EPN-RELA is more frequently observed in children and has worse prognosis. The major treatment plan for ependymoma is surgical resection plus adjuvant radiological therapies. Benefits of chemotherapy have not been reported, yet (3–6, 16–18).

#### Medulloblastoma

Medulloblastoma is the most common pediatric embryonal tumor originating from precursor cells in the cerebellum or dorsal brainstem. Like other embryonal tumors, medulloblastoma is highly proliferative and predisposed to metastasis. Histologically, medulloblastoma is classified into four different types: classic, desmoplastic/nodular, extensive nodularity and large cell/anaplastic. Medulloblastoma is one of the most heterogeneous brain tumors and currently has the best characterized molecular features. There are four distinct subgroups: wingless/integrated (WNT), sonic hedgehog (SHH), Group 3, and Group 4, which have different genetic alterations, phenotypes and prognostics.

The WNT subgroup accounts for around 10% of medulloblastoma; it mostly occurs in older children. The most common mutation in this subgroup is in the *CTNNB1* gene, which encodes β-catenin, a major player in cell cycle control and embryogenesis. The overexpression of nuclear β-catenin is often used as a diagnostic indicator in this subgroup. Monosomy chromosome 6 is another hallmark of the WNT subgroup, occurring in ~80-85% of patients, usually in conjunction with *CTNNB1* mutations. *DDX3X, SMARCA4*, and *TP53* mutations also have been reported in WNT-activated medulloblastomas (3–6, 19).

The SHH subgroup represents approximately 30% of medulloblastoma. SHH-activated medulloblastoma is highly heterogeneous and many key molecules in the SHH signaling pathway such as SUFU, smoothened (SMO), PTCH1, GLI1 and GLI2 have been dysregulated in this subgroup. Besides those, other genetic aberrations, like MYCN amplification or TP53 mutation, are also involved in SHH-activated medulloblastoma formation (19, 20). The outcome varies in SHH-activated medulloblastoma and although metastasis is not common, if a patient has a metastatic tumor, the outcome is usually worse. Moreover, patients with TP53 mutations or MYCN amplification usually have a poorer prognosis. The ongoing therapies using small molecule inhibitors target almost all affected molecules in the SHH pathway (3–6, 13, 19).

Group 3 composes around 25% of medulloblastoma. It is the most aggressive form and metastasis is very common in this subgroup. Unlike WNT- and SHH-activated medulloblastoma, the Group 3 tumors are less defined; some studies showed MYC amplification leading to tumor formation in this subgroup. Other possible pathways, such as TNFβ, have been found in around 20% of Group 3 medulloblastoma. The prognosis is overall poor for this subgroup, especially for patients with MYC amplification (4–6).

Group 4 is the most prevalent subgroup, comprising approximately 35% of medulloblastoma. Like Group 3, it has not been biologically characterized. The loss of chromosome 8, 11 and 17p or gain of chromosome 7 and 17q have been identified in this subgroup. In addition, amplification of CDK6, MYCN and SNCAP1 as well as aberrant ERBB4-SRC signaling and nuclear factor kappa B (NF-κB) have also been observed in Group 4 medulloblastoma (3–6, 19, 21, 22).

### *In Vivo* Brain Tumor Models

Most animals rarely develop spontaneous brain tumors (23) and they can be used to generate experimental models for brain tumor studies (24). A good animal model should have high incidence rate, can recapitulate original tumor’s histopathological and molecular features, and can manifest the human response to drug treatment. Numerous animal brain tumor models have been developed so far. These models can be used to investigate biological mechanisms of brain tumors and their microenvironment and for preclinical testing of novel, promising therapeutic regimens. To date, most animal models are generated with rodents, and in this review we will focus on rat and mouse models of brain tumors and discuss zebrafish brain tumor models. There are three major methods to generate animal models in brain tumor research: carcinogen induced animal models, xenograft animal models and genetically engineered animal models (23–26).

#### Carcinogen-Induced Brain Tumor Models

Rats are widely used when generating a carcinogen-induced brain tumor model, since the tumor induction in rat strains is much more effective than in mice (23). The most common carcinogens used to generate animal brain tumors are chemical carcinogens and viruses. N-nitrosourea and its derivatives have been reported to induce most common gliomas in rats, including astrocytoma, oligodendroglioma, and ependymal tumors. The embryos are much more susceptible to the chemical carcinogens, and transplacental injection is often used to administer the chemical compounds to pregnant animals (24). Injection of ethylnitrosourea to pregnant rats at gestational day 20 induced brain tumors in all of 25 pups born (27). Chemical carcinogens can also be applied to rodents through oral, intravenous or local exposure after they are born, but repeated administration might be necessary to increase induction efficacy, especially when working with older animals (23–26). The cell lines established from these chemical induced glioma models include C6, 9L, T9, F98, RG2, BT4C and CNS-1 and have been widely used in brain tumor studies (28–31). In addition to chemical carcinogens, oncogenic viruses may also be used to induce brain tumors. Both RNA viruses, such as Rous sarcoma virus-1 (RSV-1) and DNA viruses, such as adenovirus can induce brain tumors. Intracerebral injection of RSV caused malignant brain tumors in newborn pups (32). Different injection sites caused distinct tumor types (33). It has also been reported that injection of human adenovirus 12 virus (AD12) into mouse brain induced medulloblastoma or glioblastoma (34). These chemical carcinogens and oncogenic viruses are prevalent in the human environment; thus, this model can imitate natural tumorigenesis especially when animals are exposed in early development. The induced tumors can be continuously passed in animals and retain relatively stable biological characteristics. However, carcinogen induced tumor models lack consistency in tumor types, locations and biological characteristics. Moreover, the induced brain tumors are histologically and biologically different from human tumors.

#### Xenograft Models of Human Brain Tumors

Xenograft models are usually made by transplanting established cancer cell lines or brain tumor tissues derived from patients (patient-derived xenograft, PDX) or animal models into host animals. The established cancer cell lines grow very fast *in vitro* with well-defined biological characteristics, which makes them applicable to generate xenograft models. The cell lines generated from carcinogen-induced rodent tumors or from transgenic mice can be cultured and transplanted into syngeneic hosts with competent immune system (35). However, the cancer cell lines are a homogenous population lacking tumor heterogeneity and the induced tumors are rarely infiltrative. Moreover, cancer cell lines will gradually lose the original tumor phenotypes and genetic features during *in vitro* culture. Patient tissues can also be dissociated and cultured in neurobasal serum-free medium. This selects highly tumorigenic subpopulations with stem cell-like characteristics that can be grown as neurospheres before implantation into host mice (23, 24, 35, 36). In addition, the tumor tissues derived from patients can be directly transplanted into recipient animals without *in vitro* culture. Engraftments grown in these animals include tumor tissues as well as their surrounding stroma in early passage. They retain histological and molecular characteristic of original tumors, interaction between tumor and host, and a tumor’s responses to drug treatment. With this they are a more representative and reliable *in vivo* brain tumor model than those generated from cultured cells (23, 25).

In most cases to generate PDX, host animals are immunodeficient mice. The early xenografts were transplanted into nude mice, which are the first generation of immunodeficient mice. Nude mice not only lack body fur but also have no thymus. Thus, these mice have a defective adaptive immune response as they do not have T lymphocytes. Nevertheless, they still have functional B and NK cells, and an intact innate immune response causes a low engraftment rate in these mice. Later, the severe combined immunodeficient (SCID) mice that lack both functional T and B lymphocytes were generated. The engraftment efficacy has improved on SCID mice, but these mice still have remnant NK cells, hindering the engraftment rate. To eliminate the effect of NK cells, SCID mice were crossbred with Beige mice to establish SCID/Beige mice that have severely impaired NK cells and macrophages, and no mature T and B lymphocytes. SCID/Beige mice display a better engraftment rate, leading to more feasible PDX models. Since then, more immunodeficient mice strains have been established to improve engraftment and increase the success rate of PDX, such as non-obese diabetic (NOD)/SCID mice and its derivative mice (NOG, NSG and NOJ), and BALB/c background immunocompromised mice (BRG and BRJ) (37, 38). Different immunocompromised mouse strains have various sensitivity to chemotherapy or radiation, which needs to be considered when choosing an appropriate animal model. For example, BALB/c mice are very sensitive to radiation and SCID mice are sensitive to γ-irradiation and thus are not useful for radiotherapy related studies (39). Immunodeficient mice can also be modified by receiving human bone marrow to reconstitute a human immune response. These humanized mice provide an opportunity to even more closely recapitulate human brain tumors, to study the effect of the immune system on brain tumor pathogenesis, and to evaluate immunotherapies (24, 38, 39).

Xenografts can be administrated in two different ways: heterotopic xenograft and orthotopic xenograft. Heterotopic xenografts, which most typically are achieved through subcutaneous injection, are a popular method in cancer research. They are simple and convenient to observe and monitor tumors and to evaluate drug efficacies by measuring the tumor volume. However, the microenvironment of tumors induced in this way is different from the original tumor and it cannot faithfully recapitulate the original tumor initiation and progression. In addition, there is no blood brain barrier around these subcutaneous tumors, so this model cannot accurately reflect the anti-cancer drug efficacies. Orthotopic xenografts usually apply tumor cells/or tissues to the location where the original tumor is found in patients. Orthotopic xenografts can better mimic the original tumor pathogenesis, retain histological and molecular characteristics of original tumors as well as tumor host interactions (23, 25, 38, 39). However, even orthotopic xenografts may not completely maintain the histological characteristics of human tumors. Some intracranial glioblastoma xenograft models lack necrotic features and fail to show endothelial proliferation (40). To date, most available pediatric brain tumor PDX models represent glioblastoma, diffuse midline glioma, ependymoma, and medulloblastoma. The establishment of PDX models for less aggressive brain tumors, such as pilocytic astrocytoma, has been less successful due to a very low tumor engraftment rate (39).

The development of pediatric brain tumor PDX models emerged over thirty years ago (24, 38). In recent years, with the raised interest in some pediatric brain tumor types and increased availability of tumor tissues, more and more PDX models have been generated. In 2018, a brain tumor biology study sponsored by the Children’s Oncology Group led to generation of 30 orthotopic pediatric brain tumor PDX models, including medulloblastoma, high grade glioma and ependymoma. These PDX models are valuable tools to investigate subtype specific pediatric brain tumors, since they preserve the original tumors’ histological and molecular features and remain relatively stable when being passaged in mice (41). The scientists from St. Jude Children’s Research Hospital also successfully generated 37 novel orthotopic PDX models derived from pediatric brain tumor patients including 22 medulloblastomas and 5 ependymomas, which also maintain original tumors’ histological features and are genetically faithful to corresponding patient tumors (42). The Mayo Clinic Brain Tumor Patient-Derived Xenograft (PDX) National Resource has also established a repository of glioblastoma PDX models with highly characterized molecular subtype and phenotype. Another study collected DIPG samples from patient autopsies and biopsies at 8 different international institutions and generated 22 *in vivo* xenograft models, covering the main molecular subtypes including H3.3 K27M and H3.1 K27M mutations (43, 44).

With the advance in gene editing technology, neural stem cells (NSC) can be genetically engineered to acquire tumorigenic capability and used to generate xenograft models (35). Transplantation of NSCs that overexpressed *myc* alone, or with oncogene *gfi1* or *gfi1b* into the cerebella of immunocompromised mice induced Group 3 medulloblastoma (45–48). Funato et al. successfully transformed neural progenitor cells derived human embryonic stem cells with a constitutively active form of the PDGFRA, a small hairpin RNA (shRNA) against p53 and H3.3K27M to model pediatric DIPG with H3.3K27M mutation (49). In addition, NSCs co-expressing PDGFRB and H3.3K27M were injected into the pons of SCID mice and induced tumors similar to human H3K27M DIPGs (50). The first mouse model of ependymoma was generated by implanting embryonic cerebral *Ink4a/Arf*−/− NSCs overexpressing Ephb2 into the cerebrum of immunocompromised mice (51). More recently, induced pluripotent stem (iPS) cells are also being used to generate brain tumor xenograft models. iPS-derived neural stem cells generated from Gorlin syndrome patients, who are carrying a germline mutation in PTCH1 and are predisposed to medulloblastoma, were transplanted into mouse cerebellum. These cells formed tumors that mimic SHH-driven medulloblastoma (52, 53).

Xenograft models are a valuable tool in cancer research and drug screening. The National Cancer Institute recently decided to use PDX models to replace a panel of 60 human cancer cell lines (NCI-60) as a model for drug screening (54). However, there are some limitations of xenograft models. First, the generation of some xenograft models is challenging, but the success rate is increased with more aggressive and highly malignant tumors. Second, xenografts usually require many cells at a time, which is not naturally occurring in patients. Third, the transplantation procedure can disrupt the blood brain barrier, which is a key factor when evaluating drug efficacy. Fourth, the host animal for xenografts are usually immunodeficient mice, which cannot be used to discover the contribution of immune system in tumor initiation and development. Fifth, the engrafted human tumor stroma structure will be lost over time, replaced by the host mice’s own microenvironment. Sixth, genetic and phenotypic drifts gradually occur as the xenograft tumors are propagated through mice. Last, maintenance of PDX models is costly and labor intensive (22–25, 35, 38, 39, 55).

#### Mouse Models With Genetic Engineering of Brain Tumors

In recent years, with rapid advances in gene editing techniques, genetically engineered mouse models (GEMMs) have gained popularity in brain tumor research. Unlike PDXs, GEMMs can recapitulate tumor initiation and development in animals with native immune system and intact blood brain barrier and undisrupted microenvironment. This makes GEMMs more attractive as models for tumor mechanism and drug discovery studies (23, 24, 26, 56). Moreover, other genetically engineered animal models, such as mice expressing enhanced green fluorescent protein (EGFP) or humanized mice carrying human functional biological system are valuable tools in brain tumor research, especially in studies about tumor host interactions and human-specific pathogenesis and therapies (57, 58).

GEMMs for cancer research can be generated by introduction of oncogenes or disruption of tumor suppressor genes in embryonic stem (ES) cells or zygotes (25) and include both transgenic mice and knockout mice. In addition to oncogenes and tumor suppressor genes, key molecules in tumor signaling pathways can also be utilized to develop GEMMs (**Table 1**). The conventional knockout models alter target gene expression in all tissues throughout the whole mouse. These constitutive changes often lead to more severe phenotypes with contribution by the brain tumor itself as well as other conditions. This makes data analysis and interpretation more difficult and less accurate. In some cases, this global knockout can even be lethal in animals. To overcome this issue, conditional or inducible conditional knockout has been developed, in which the target gene can be edited in a tissue specific and/or time-dependent way. The most common tool to make conditional knockout mice is the Cre-loxP system. Cre is a recombinase and its expression can be driven under the control of a tissue-specific promoter. When Cre is induced, it can recognize the loxP sites and catalyze the recombination, so the target gene flanked with two loxP sites in the same orientation will be excised. To achieve precise temporal specificity in the Cre-loxP system, Cre can be fused with a hormone responsive element, and induced by the exogenous inducers tamoxifen or tetracycline (35, 106).

**Table 1:** The common GEMMs of pediatric brain tumors.

Tumor type	Molecular subtypes	Mouse model name	References
Medulloblastoma	WNT	*Blbp-Cre*^+/−^: Ctnnb1^+/lox(ex3)^; Tp53^flx/flx^	(59)
		*Blbp-Cre*^+/−^; *Ctnnb1^+/lox(ex3)^*; *Tp53^+/flx^*; *Pik3ca^loxE545K/loxE545K^*	(60)
	SHH	Ptch1^+/-^	(61)
		Ptch1^+/−^; Math1-Cre	(62)
		Ptch1^+/−^; hGFAP-Cre	(62)
		Ptch1^+/−^; Math1-CreER	(63)
		Ptc1^+/−^; p53^−/−^	(64)
		Ptc1^+/-^; Ink4c^-/-^	(65)
		Ptc1^+/−^; Kip1^−/−^	(66)
		Ptch1^+/−^; Hic1^+/−^	(67)
		Ptch1^+/-^; Ptch2^-/-^	(68)
		NeuroD2-SmoA1 (W539L)	(69)
		Smo/smo (homozygous smoA1)	(70)
		NeuroD2-SmoA2 (S537N)	(71)
		*CAGGS-CreER; R26-SmoM2*	(72)
		p53^-/-^; Sufu^+/-^	(73)
		Trp53^−/−^; PTEN^−/−^	(74)
		Trp53^−/−^; Parp^−/−^	(75)
		Nestin-tv-α mice infected with RCAS-Shh + N-Myc	(76)
		Nestin-tv-α mice infected with RCAS-Shh + N-Myc (T50A)	(76)
		Nestin-tv-α mice infected with RCAS-Shh + Bcl2	(77)
	Group3	Gtl1-tTA : TRE-MYCN/luciferase (GTML)	(78)
		GTML; Trp53^-/-^	(79)
		Mll4^-/-^; Nestin-Cre	(80)
		Nestin-tv-α; Trp53^-/-^ mice infected with RCAS-Myc	(81)
		Nestin-tv-α mice infected with RCAS-Myc + Bcl2	(81)
		Co-electroporation of Myc and trp53DN into embryonic cerebellar progenitor cells	(82)
	Group4	Co-electroporation of SRC-CA and DNp53 into E13.5 developing cerebella	(83)
Gliomas		Nf1^+/-^; p53^+/-^	(84)
		*p53−/−;NF1flox/flox;hGFAP-cre+*	(85)
		*cisp53+/−;NF1+/flox;hGFAP-cre+*	(85)
		*cisp53+/−;NF1+/flox; Pten*^f/+^;*hGFAP-cre+*	(86)
		*TgGFAPT121*	(87)
		GFAP-V^12^Ha-ras	(88)
		GFAP-V^12^Ha-ras;GFAP-EGFRvIII	(89)
		GFAP-V^12^Ha-ras*;Ptenf/f*; hGFAP-Cre	(90)
		S100β-*v-erbB*	(91)
		S100β-*v-erbB*; Ink4a/Arf^-/-^	(91)
		S100β-*v-erbB; p53 +/-*	(91)
		Nestin-tv-α mice infected with RCAS K-ras and Akt	(92)
		Nestin-tv-α; *PtenloxP/loxP* mice infected with R*CAS KRAS and* RCAS cre	(93)
		Nestin-tv-α mice infected with RCAS–PDGF-B	(94)
		GFAP-tv-α mice infected with RCAS–PDGF-B	(94)
		Nestin-tv- α mice infected with RCAS-PDGFB	(95)
		Nestin-tv-a; Ink4a-arf^−/−^ mice infected with RCAS-PDGFB	(95)
		Nestin-tv-a; p53^fl/fl^ mice infected with RCAS-PDGF-B and RCAS-Cre	(96)
		GFAP tv-a; p53^fl/fl^ mice infected with RCAS-PDGFB + RCAS Cre	(97)
		Nestin tv-a; p53^fl/fl^ mice infected with RCAS-PDGFB+RCAS-Cre	(97)
		Pdgfra (D842V); Trp53 (R270H); H3f3a (G34R) (MDRA mice)	(98)
		Pdgfra (D842V); Trp53 (R270H); H3f3a (K27M) (MDRA mice)	(98)
		Erbb2-V664E; *PiggyBac* transposon	(99)
		Hras-G12V; *PiggyBac* transposon	(99)
		Kras-G12V; *PiggyBac* transposon	(99)
		Pdgfra-D842V; *PiggyBac* transposon	(99)
		PBCAG-Ngn2/PBCAG-HRasV12/Akt; in utero *Piggy Bac* transposon in different cell lineages *	(100)
		PBCAG-NeuroD1/PBCAG-HRasV12/Akt; in utero *Piggy Bac* transposon in different cell lineages *	(100)
		PTEN; NF1; P53 PiggyBac transposon-CRISPR/Cas9*	(101)
Ependymoma	ST-EPN-RELA	Nestin-tv- α mice infected with RCAS-*RELA^FUS1^*	(102)
	ST-EPN-RELA	GFAP-tv- α mice infected with RCAS-*RELA^FUS1^*	(102)
	ST-EPN-RELA	BLBP-tv- α mice infected with RCAS-*RELA^FUS1^*	(102)
	ST-EPN-YAP1	YAP1-MAMLD1	(103)
	ST-EPN-YAP1	LATS1^f/f^ LATs2^f/f^: NEX^Cre/+^	(104, 105)
	ST-EPN-YAP1	nlsYAP5SA/+: NEX^Cre/+^	(104, 105)

*denotes a rat model.

The generation of germline GEMMs usually needs an extensive breeding scheme, which is time-consuming and expensive. Thus, virus mediated gene transfer is introduced to deliver Cre recombinase to somatic cells to establish non-germline GEMMs, which retains the ability of spatial and temporal gene regulation, at the same time also reduces the cost and time by bypassing complicated breeding (25). Replication-competent avian sarcoma-leukosis virus long terminal repeat with splice acceptor/tumor virus A (RCAS/TVA) is a commonly used system. RCAS is a retrovirus that enters specific cells *via* binding to its specific cell surface receptor TVA. TVA is only expressed in avian cells, but mammalian cells can gain the expression through genetic engineering (107). RACS/TVA based GEMMs have some advantages over Cre-loxP based models. The virus transduction rate is quite low, so only a small fraction of cells can acquire the expression of target genes. This makes the model close to natural tumorigenesis, since studies have shown that only small amounts of cancer stem cells are key players in tumor initiation (35). Moreover, genetically-engineered mammalian cells can get multiple RACS infection simultaneously or sequentially, which makes this model suitable to study the effect of multiple genes on tumorigenesis (107).

Recently, short palindromic clustered regularly interspaced repeats/CRISPR associated protein 9 (CRISPR/Cas9) technology has become a powerful tool to generate GEMMs. The CRISPR/Cas9 system is a groundbreaking gene editing technique; it consists of two necessary components: single strand guide RNA, which can recognize the target genomic DNA sequence, and endonuclease cas9, which can break the double-stranded DNA at the target sequence site. Then random or targeted gene editing can be achieved by DNA repair through error-prone non-homologous end joining (NHEJ) and high-fidelity homology directed repair (HDR) pathways. CRISPR/Cas9 can efficiently introduce gene modification on virtually any genetic background, both in germline and somatic cells. It turns conventionally tedious and expensive genetic engineering into a simple, fast and affordable procedure and dramatically broadens the application of GEMMs in tumor research (35, 108).

In 2019, MADR (mosaic analysis by dual recombinase-mediated cassette exchange) was introduced as a simpler, higher-throughput method to generate stable, defined copy number somatic transgenic animals. MADR was designed to overcome limitations of some of the previously described methods to generate mouse models (98). This includes the limited payloads and possible immune reactions when using viruses, the unpredictable genomic integration patterns, epigenetic transgenic silencing, transgene copy number variability, and overexpression artifacts such as cytotoxicity and transcriptional squelching when using viruses or transposons, or the variability and unintended off-target genomic alterations of CRISPR/Cas9 systems (98).

To date, the majority of pediatric brain tumor GEMMs are SHH-activated medulloblastoma (**Table 1**), which are generated by modifying SHH signaling genes, such as *PTCH*, *SMO*, or *SUFU* (22, 24). The first mouse model of medulloblastoma was established by disrupting *ptch1* (61). Thereafter, the combination of a *ptch* mutation with inactivation of tumor suppressors, including TP53 or cyclin D-dependent kinase inhibitor p18^Ink4c^, was used to generate different SHH driven medulloblastoma models with shorter latency and higher penetrance (22, 55, 109). SHH medulloblastoma models were also developed by overexpressing Shh, alone or in combination with *mycn* or *bcl2* with the RACS-TVA system (76, 77). Most WNT activated medulloblastoma models were generated by targeting the gene *ctnnb1* in progenitor cells of the dorsal brainstem. However, *ctnnb1* aberration alone was not sufficient to form medulloblastoma and the combination with TP53 mutation was needed to drive tumor initiation (59). Co-occurrence of *pik3ca* mutation significantly accelerated the formation of WNT medulloblastoma and dramatically increased tumor penetrance in mice (60). Most GEMMs of Group 3 medulloblastoma were developed by targeting *myc*. Newborn mice with *myc* overexpression in the cerebellum through the RCAS/TVA system induced Group 3 medulloblastoma, but tumor formation required *tp53* loss or *bcl-2* overexpression (81). Conditional enforced co-expression of *myc* and a dominant-negative form of *Trp53* (*Trp53DN)* in embryonic cerebellar progenitor cells by in utero electroporation also induced Group 3 medulloblastoma in mice (82). The first mouse model of Group 4 medulloblastoma was recently developed by overexpression of an activated SRC combined with p53 inactivation in the developing cerebellum (83). It is worthy of note that in some animal models induced tumors simultaneously possess multiple molecular characteristics. For example, the GTML (Glt1-tTA/TRE-MYCN-Luc) model in which MYCN aberration is driven by the glutamate transporter 1 (Glt1) promoter expressed in hindbrain progenitors develops tumors that closely resemble Group 3, but also shows the features of WNT, SHH, and Group 4 medulloblastoma (24).

Various medulloblastoma mouse models have been used to dissect mechanisms, including those influenced by the tumor microenvironment, that regulate the progression from precancerous lesions to medulloblastoma tumors (110). In general, tumors not only consist of the heterogenous tumor cell population but also of the extracellular matrix (ECM) surrounding the cells, resident and infiltrating cells such as tumor-associated fibroblasts, endothelial cells, pericytes, adipocytes, and immune cells including lymphocytes and macrophages as well as soluble factors, including cytokines, chemokines, growth factors, matrix remodeling enzymes and inflammatory enzymes (111, 112). The tumor microenvironment is known to contribute to tumor progression, metastasis formation and therapeutic response (113–122). In brain tumors, macrophages are the most abundant type of immune cells and are particularly high in Shh-driven medulloblastoma. In humans, decreased macrophage numbers are correlated with significant poorer outcome and indeed, a recent study in *NeuroD2:SmoA1* mice and derivative mouse lines was able to demonstrate that tumor-associated macrophages have properties that kill tumor cells (123). The *NeuroD2:SmoA1* medulloblastoma model was also used to show that blocking TGF-β signaling promoted memory T cell development thereby conferring antitumor immunity (124) and in *Atoh1-Cre;Ptch1^fl/fl^* mice, tumor astrocyte-derived Shh induced the proliferation of medulloblastoma tumor cells (125). Cancer stem cells reside in specialized, anatomically distinct niches within the tumor microenvironment (126) and medulloblastoma stem cells (Nestin^+^, Prominin^+^) are closely associated with capillaries in the perivascular niche. Using mice infected with RCAS-Shh RCAS-SHH in combination with RCAS-N-myc-T50A or RCAS-AKT-Myr Δ11–60 of Ntv-a wild-type p53 and Ntv-a p53-null background, Hambardzumyan et al. showed that similar to human medulloblastomas, nestin-expressing perivascular stem cells survive radiation, activate PI3K/Akt signaling, undergo PTEN/p53-dependent cell cycle arrest and shortly thereafter re-enter the cell cycle (127). Medulloblastoma mouse models have also been used to elucidate pathways involved in tumor angiogenesis, in medulloblastoma metastasis, and in cell senescence and reprogramming (110).

Most mouse models of glioma are generated by altering key signaling pathways disrupted in human gliomas, including Ras, EGFR, Akt, Rb, Pten, Nf1 and platelet-derived growth factor (PDGF) (**Table 1**). GFAP-V12Ha-ras mice were generated by overexpressing oncogenic V12Ha-ras in astrocytes, and 95% of these mice died from low- and high-grade astrocytoma within 2-6 months (88). Further expressing a mutant EGFRVIII or inactivating PTEN in GFAP-V12Ha-ras mice demonstrated earlier tumor onset, higher tumor grade and a dramatic reduction in survival (89, 90). Introduction of activated Ras (KRas) into neural progenitors with the RCAS/TVA system, combined with activated Akt or PTEN loss induced high-grade gliomas in mice that resembled human GBMs (92, 93). Further deleting ink4a/arf increased tumor incidence and grades in these mice (128). Silencing of Bcl6 in neuronal precursor cells suppressed, but did not abolish, the formation of tumors in a somatic KrasG12V-driven glioma mouse model (99, 129). Transgenic S100β-v-erbB mice in which a transforming allele of EGFR, v-erbB, is expressed under the control of murine S100β promotor developed low-grade oligodendroglioma, and further deleting *ink4a/arf* or *p53* increased tumor grade and penetrance (91). An Nf1+/-; p53+/- mouse model shows a range of astrocytoma stages, from low-grade astrocytoma to glioblastoma multiforme (84). Conditionally deleting NF1 in glial progenitors and astrocytes of p53 null mice dramatically increased the penetrance of induced astrocytoma and the incidence of non-CNS neoplasms (85). Further loss *of Pten* in glial progenitors and astrocytes of this mouse model significantly accelerated tumor growth and animal mortality (86). TgGFAPT121 mice generated by a truncated SV40 T antigen (T121) to inactivate the Rb pathway in astrocytes develop high grade astrocytoma and die perinatally (87). PDGF B-chain (PDGF-B) is another common target used to generate glioma models. PDGF-B delivered to nestin-positive neural progenitors or GFAP positive astrocytes induced low grade glioma in mice. Loss of *Ink4a–Arf* dramatically shortened tumor latency and enhanced malignancy of gliomas. p53 loss can also enhance PDGF-B driven glioma in mouse models (94–96). A more recent DIPG model was developed by overexpressing PDGF-B and H3.3K27M together with p53 loss in nestin-positive neural progenitors. The induced tumors in the brainstem of these mice demonstrated DIPG-like features that recapitulate the histopathological and molecular characteristic of human DIPG (96, 130). The MADR method was used to generate pediatric glioma mice modeling simultaneous H3f3a, Pdgfra, and Trp53 mutations with two missense mutation variants G34R or K27M that recapitulated human tumor heterogeneity and developmental hierarchy (98). Other recent pediatric brain tumor models have also been successful in capturing tumor heterogeneity and spatiotemporal characteristics of pediatric gliomas. *PiggyBac* transposon systems not only can circumvent the loss or inactivation of episomal plasmids delivered to glial cells *via in utero* electroporation but also allow for expression of multiple oncogenes in selected cell populations at different times in brain development (131). Using in utero electroporation of *piggyBac* transposons, Chen and colleagues generated rat tumor models by directing HRasV12 and AKT to different cell populations. Using the same transgene under the control of different promoters resulted in tumors ranging from glioblastoma multiforme to anaplastic oligoastrocytomas and atypical teratoid/rhabdoid-like tumors that could be distinguished at the cellular and the molecular level (100, 131). Moreover, targeting different genes, PTEN or NF1, in the same lineage resulted in distinct neuropathologies and when PTEN, NF1 and P53 were targeted simultaneously caused the formation of GBM (101).

The generation of ependymoma GEMMS began recently. RELA^FUS^1 fusion gene expressed in nestin, GFAP, or BLBP positive cells in the mouse brain induced tumors which recapitulate the histology and transcriptome panel of human ST-EPN-RELA ependymomas (102). The YAP1-MAMLD1 fusion gene delivered to mice by in utero electroporation drove tumor formation and tumors share histological and molecular characteristics of human ST-EPN-YAP1 (103). Recently, Eder and colleagues reported that ectopic expression of active nuclear YAP1 (nlsYAP5SA) or conditional deletion of YAP1**’**s negative regulators LATS1 and LATS2 kinases in neural progenitor cells in ventricular zone also induced tumors which display molecular and ultrastructural characteristics of human ependymoma (104, 105).

#### Zebrafish Brain Tumor Models

Zebrafish are an alternative model to study human cancer as they can develop tumors that are histologically and genetically similar to those in humans (132). Zebrafish models are also amenable to high-throughput screening for drug discovery as well as transplantation of primary patient tumors. This makes zebrafish a cost- and time-effective alternative to other *in vivo* tumor models such as rodents. In recent years, several pediatric brain tumor models have been developed in zebrafish and have been used to identify molecular mechanisms driving tumor formation. This includes the analysis of unique and shared molecular pathways driving pediatric HGG within and outside the brainstem (133) and to identify three molecular subgroups of DIPG (134). Ependymoma, glioma and choroid plexus carcinoma cells from mouse models of pediatric brain tumors were conditioned to grow at 34°C and used for orthotopic xenografts in zebrafish. These cells not only readily formed tumors but also spinal metastasis. The tumors retained the histological characteristics of the corresponding mouse tumor and formed tumor vasculature by recruiting fish endothelial cells (135). Lin et al. used zebrafish to experimentally validate subgroup-specific enhancers in medulloblastoma (13), Modzelewska et al. used tumors grown in zebrafish to demonstrate that MEK inhibitors can reverse the growth of embryonal brain tumors derived from oligoneural precursor cells (14) and Idilli et al. used them to study telomere maintenance mechanisms in pediatric brain tumors (136). With protocols for developing zebrafish tumor models evolving, long-term orthotopic transplantation of tumor cells is now possible (137). This allows for the long-term *in vivo* studies of tumor cell behaviors including tumor invasion and dissemination as well as testing for more durable response of tumors to novel anticancer therapeutics and the development of cancer drug resistance. Overall, zebrafish may provide an opportunity to develop pediatric brain tumor models in a timely and affordable manner for preclinical drug discovery in a model system with intact blood-brain barrier.

### *In Vitro* Brain Tumor Models

#### Cancer Cell Lines

Cancer cell lines play an important role in brain tumor research. The cells lines can be established directly from patients’ samples or from animal models. These cells often retain original tumor features, are easy to grow and propagate, and can be stored for a long time. They are well-suited models to explore a tumors’ molecular features *in vitro* and predict the tumors’ response to therapeutic regimens. Cancer cell lines are particularly useful in high-throughput drug screening to identify and evaluate potential targets for chemotherapies. Most established pediatric brain tumor cell lines are medulloblastoma cell lines. Less than half of these cell lines have been molecularly defined, among which the majority represent the SHH or Group 3 subtypes; only a few are for WNT or Group 4 tumors (**Table 2**). In addition, around half SHH medulloblastoma cell lines have mutations in *TP53*, and almost all Group 3 cell lines bear *MYC* amplification, while only a small part of SHH and Group 3 medulloblastoma patients typically have these mutations (24, 55, 145, 150). This discrepancy might be because the medulloblastomas with TP53 mutation and MYC amplification are more aggressive with poorer prognosis, and more aggressive cells are easier to grow *in vitro*. Similarly, although gliomas are the most common brain tumors in children, most gliomas are low grade gliomas with less malignancy and more favorable prognosis. Moreover, some high grade gliomas, such as DIPG, have a limited tissue availability due to tumor locations and established glioma cell lines are fewer than medulloblastoma (150). In recent years, however, with the refinement of surgical skills and advances in DIPG biology, biopsy becomes more feasible in DIPG patients and new patient derived DIPG cell lines are becoming available (**Table 2**).

**Table 2:** Established pediatric brain tumor cell lines with defined molecular characteristics.

Tumor Type	Molecular subtype	Cell line name	Mutations	Sources	References
Medulloblastoma	WNT	MED5R	β-catenin		(138)
	SHH	DAOY	CDKN2A NF1 TP53	ATCC	(139)
		ONS76		JCRB	(139)
		UW228	TP53		(139, 140)
		UW426			(139)
	Group 3	D341 Med	myc amplification	ATCC	(141, 142)
		D384 MED	myc amplification		(143)
		D425 MED	myc amplification p53	Millipore Sigma	(144)
		D458	Myc amplification		(145)
		D283 Med	myc amplification	ATCC	(144, 146)
		MED8A	myc amplification		(147)
		HD-MB03	myc amplification	DSMZ	(148)
		MB002	myc amplification		(149)
		Med-114FHTC	myc amplification	BTRL	https://www.btrl.org/product/med-114fhtc/
		Med-411FHTC	myc amplification Isochromosome 17	BTRL	https://www.btrl.org/product/med-411fhtc/
		Med-2112FHTC	myc Isochromosome17	BTRL	https://www.btrl.org/product/med-2112fhtc/
	Group 4	CHLA-01-MED	Myc amplification	ATCC	(150)
		CHLA-01R-MED	Myc amplification	ATCC	(150)
High grade glioma	MYCN	PBT-04FHTC	mycn, id2, nras	BTRL	(41)
		PBT-05FHTC	mycn, id2, egfr amplification	BTRL	(41)
	pedRTK1	GBM-511FHTC	cdkn2	BTRL	(41)
	pedRTK2	GBM-110FHTC	cdkn2, braf	BTRL	(41)
	Myc	CHLA-200	myc	COGcell.org	(151)
DIPG	H3.3 K27M	SF7761	Histone	Millipore Sigma	(152, 153)
		SF8628	Histone	Millipore Sigma	(153)
		PED8	Histone		(130)
		PED17	Histone		(130)
		PED36	histone		(130)
		HSJD-DIPG-007	histone		(50)
		HSJD-DIPG012	histone		(50)
		HSJD-DIPG017	histone		(50)
		SU-DIPG-VI	histone		(154)
		SU-DIPG-XIII	histone		(154)
		VUMC-DIPG-A	histone		(154)
		JHH-DIPG-1	histone		(154)
	H3.1 K27M	VUMC-DIPG-B	histone		(154)
		SU-DIPG-IV	histone		(154)
		HSJD-DIPG018	histone		(50)
GBM	H3.3 G34R	GBM002	histone		(50)
	H3K27M	GBM003	histone		(50)
Ependymoma	PF-EPN-A	EPD-210FHTC	1q gain	BTRL	(41)
		EPN-811	1q gain		(155)
		EPN-928	1q gain		(155)

Cell culture *in vitro* has intrinsic drawbacks. Most cell lines are maintained as a monolayer culture in serum containing media, and a genetic and phenotypic drift from original tumors will gradually occur with passage (156). The cell lines are homogenous populations that cannot fully recapitulate the heterogeneity of tumors, and are not suitable to study tumor host interactions during tumor development. In monolayer culture, all cells receive the same level of nutrition and oxygen, which is different from tumor growth *in vivo*. Moreover, tumor cell lines are typically grown on borosilicate glass or clear plastics *in vitro*, which are much more rigid compared to extracellular matrix on which cells are naturally grown *in vivo* (157).

The advent of neurosphere cultures addressed some limitations of traditional cell cultures. Neurospheres are typically cultured in serum-free medium and can maintain tumor heterogeneity and preserve the phenotype and genotype of primary tumors (158). Some pediatric brain tumors, including DIPG, have been successfully cultured in neurospheres and used to generate xenografts that recapitulate the histological features and infiltrative growth of original patient tumors (24, 130, 152, 159). Neurosphere formation also can independently predict clinical outcome in malignant glioma (160). Therefore, neurospheres are a more representative and reliable cell model compared to traditional cell lines (36). However, neurosphere culture has some limitations, too. For example, the lack of a tumor microenvironment highly enriches glioma stem cell (GSC)-like cells, which only represent a relatively small subpopulation in native tumors (158, 161).

#### Three-Dimensional Culture

3D culture such as spheroids and scaffold-based cultures are other techniques that have been developed to overcome the limitations with traditional monolayer culture. In scaffold-based 3D cultures, extracellular matrix can be synthesized to simulate a tumor’s natural microenvironment and a gradient of oxygen and nutrient level can be constituted to mimic a tumor’s hypoxic core *in vivo*. Moreover, gene expression panels in 3D culture more closely resemble human tumors *in vivo* (157, 162, 163). To date, there are two major types of 3D culture: anchorage-dependent and anchorage-independent 3D models.

##### Anchorage-Independent 3D Models

Anchorage-independent 3D models are achieved mainly by self-assembly of cells grown in special tissue culture plates, such as hanging drop microplates and low attachment plates; they do not need any scaffold to facilitate the culture. The hanging drop culture is a well-known 3D culture technology. Typically, there is a micro-hole at the top of wells, which allows the medium to pass through and form a small droplet. Since there is no surface available for the cells in the droplet to attach, these cells tend to form spheroids. Spheroids can also be generated when they are grown in ultra-low attachment plates. Ependymoma cell lines cultured in ultra-low attachment plates better recapitulated the histological and transcriptional features of the primary tumors when compared to a monolayer (164). In addition, magnetic levitation is a newly developed method for spheroid formation. In this method, cells coated with magnetic nanoparticles are cultured in a magnetic field and the cells are floated toward the air/liquid interface within a low adhesion plate to form spheroids (162, 163, 165). Larger tumor spheroids formed by anchorage-independent models may consist of a peripheral layer with proliferating cells, an intermediate layer with quiescent cells and an inner necrotic core, which may closer reproduce human tumor architecture *in vivo* (165, 166). In recent years, with the refinement of technologies on spheroid culture, anchorage-independent 3D models have become common methods for cancer drug discovery, even applicable for high-throughput drug screening. Spheroids can be established with a few different types of cells and are especially suitable for studies about cell-cell interactions during brain tumor development (167). However, there is typically no extracellular matrix (ECM) in spheroids, and thus they are unsuitable for studying cell-host interactions which is a key game player in tumor pathologies.

##### Anchorage-Dependent 3D Models

The cells inside the body are usually surrounded by ECM, a network of extracellular molecules, which not only provides the structural scaffold for the surrounding cells, but also plays an important role in cell proliferation, differentiation, migration, survival and adhesion (168). The composition of ECM is highly heterogeneous and tissue-specific. The brain ECM ingredients include proteoglycans, hyaluronic acids, tenascins, collagen, fibronectin, vitronectin and laminin (162, 169). In anchorage-dependent 3D models, cells are encapsulated into scaffold materials, which can mimic the composition and key physical properties of ECM. Hydrogels are the most commonly used scaffolds for anchorage-dependent 3D models. Hydrogels are water-swollen networks of polymers and can mimic salient components of ECM. The highly hydrated and porous nature of hydrogel make them ideal to encapsulate cells and render ECM-like functions, such as supporting cell survival, growth, differentiation and modulating the response to chemotherapy, immunotherapy and radiation therapy (161).

Hydrogels may come from natural sources or can be synthetic. The widely used natural hydrogels for neural cell culture are collagen I and Matrigel. Matrigel is extracted from the Engelbreth-Holm-Swarm (EHS) mouse sarcoma, a tumor rich in ECM components, such as laminin, collagen, heparan sulfate proteoglycans, entactin/nidogen, and several growth factors. Matrigel is minimally processed and it can better mimic *in vivo* ECM (170). However, two major components of Matrigel are laminin and collagen, which are in low concentration in the brain ECM (158). Thus, collagen and Matrigel are not ideal choices as *in vivo*-like 3D scaffolds for brain tumor cells. In addition, collagen and Matrigel are derived from natural sources, they are heterogenous and not well defined, and exhibit considerable batch-to-batch variability. Moreover, collagen and Matrigel are available in liquid form and require handling at cold temperatures (below 10°C) to avoid premature gelation. The need for handling these hydrogels at low temperatures makes them poorly suited for common liquid handling equipment used for high-throughput screens in drug discovery (158, 162). Some of these limitations might be overcome by synthetic hydrogels. Synthetic hydrogels are derived from polymeric materials, such as polyethylene glycol (PEG), polylactic acid (PA) and polyglycolic acid (PGA). These hydrogels can simulate the composition and function of ECM; they often have engineered tunable properties to achieve desired stiffness and porosity, to enhance cell proliferation and differentiation by encapsulating bioactive molecules, such as growth factors or hormones. However, these polymers are biological inert, so they must be modified by addition of cell adhesion ligands or mixing with other natural ECM components to acquire the properties of cell adhesion (158, 162, 163). To date, PEG is a widely used synthetic hydrogel in neural cell 3D culture. A PEG-based hydrogel has been successfully used to grow GBM cell lines. In this system, the PEG-based hydrogel was modified with CRGDS and a MMP-cleavable peptide to facilitate cell proliferation, migration; hyaluronic acid (HA) was also added to mimic brain extracellular matrix (171). A synthetic MAX8 β-hairpin hydrogel was successfully used to culture pediatric medulloblastoma cell lines in a high-throughput screening setting (172, 173). Hydrogel-based models are not suitable for long-term culture since they degrade fast. Solid porous scaffolds can be adapted to bypass this issue. Solid porous scaffolds are prepared from natural or synthetic polymers with mechanical stability and pore interconnectivity. The cells can be directly added to these solid porous scaffolds and maintain their 3D properties with continuous supply of nutrients. A recently developed tunable 3D brain tissue model integrated the porous scaffold with hydrogels. In this model, the donut shaped silk fibroin protein scaffold was infused with ECM hydrogels and brain tumor cells can grow into spheroids within the stiff silk scaffold, or migrate toward the central hydrogel. Thus, the outer-ring scaffold can be used to anchor neuronal cells, and the central soft hydrogel allows axonal penetration and connectivity (174, 175). A pediatric anaplastic ependymoma has been successfully cultured by this model (176). In addition, culturing glioblastoma tumor-initiating cells (TICs) in microscale alginate hydrogel tubes (AlgTubes) has been reported. This culture system allows for long-term and scalable production of glioblastoma cells for drug discovery (177). Self-assembling peptide (SAP) hydrogels are an evolving field for neural cell culture. These synthetic peptides can self-assemble under physiological conditions and support neural cell attachment, differentiation and synapse formation. SAP hydrogels are highly versatile, their material properties can be modulated by substituting amino acids, extending or shortening the peptide sequence, or by the addition of functional epitopes. A widely used peptide hydrogel is RADA16. However, peptide-based hydrogels may have poor mechanical properties, and some exhibit impaired cell viability caused by low pH, making it difficult to culture sensitive brain tumor cells (163).

#### Brain Organoids

Organoids are an emerging technology to study pediatric brain tumors. Organoids are typically generated with embryonic stem cells (ESC) or induced pluripotent stem cells (iPSC) and have the potential to grow into a 3D architecture in a way similar to *in vivo* tissue development by virtue of their capacity to self-renew and differentiate. Early organoid models were typically heterogeneous and lacked reproducibility since it was difficult to control the differentiation pattern of stem cells. However, with technical advances on directed differentiation, stems cells can now be differentiated into virtually any specific lineages. This has significantly moved forward the application of organoid models including in biomarker and drug discovery (163). Significant effort is being made in developing neural-based spheroids with cerebral organoids being one of the early ones (178). Cerebral organoids can be used as platforms for human brain tumor cells, or tumors can be initiated in cerebral organoids by introducing oncogenes and/or disrupting tumor repressor genes using gene editing technologies. Human cerebellar organoids derived from iPS cells electroporated with Otx2/c-MYC induced Group 3 medulloblastoma (179). Injecting cancer stem cells derived from GBM patients into cerebral organoids or genetic engineering of cerebral organoids by introducing *HRas^G12V^* and disrupting *p53* initiated tumorigenesis that closely recapitulated patient GBMs (180, 181). Organoids can also be established from patient brain tumors. Hubert et al. generated GBM organoids directly from patient samples that present hypoxic gradients and regional tumor heterogeneity (182). Organoids can theoretically resemble any *in vivo* brain niche with preserved cell distribution, can retain genetic and phenotypic stabilities, and are capable of long term culture; this makes them a valuable model to discover tumor initiation and progression, and a more accurate tool to predict the responses to tumor treatments. However, organoid cultures typically lack blood vessels and immune cells, which makes them unsuitable for testing tumor treatments targeting angiogenesis, or studying the contribution of immune system on tumorigenesis and relevant therapies. In addition, although organoids have proper cell composition and functions, they typically lack correct anatomical organization (162, 183). Another drawback of organoids and also found in spheroid models is that they often have a necrotic core, which sets a limit on the culture size and longevity. To overcome this limitation, microfluidic devices can be incorporated into 3D models. Microfluidic devices are designed for cell cultures under perfusion and allow for steady supplies of oxygen and nutrients while at the same time removing waste (163).

## Conclusions

A precise *in vivo* pediatric brain tumor model is the one, which can faithfully recapitulate tumor’s histopathological and molecular features; exhibit tumor’s spatiotemporal characterization; demonstrate a tumor’s microenvironment; predict patients’ response to treatments; show high rate of incidence and short latency; and is reproducible, timesaving and cost-effective (184). Such accuracy in tumor models can best be achieved when genetic insults match the cell of origin and are introduced at developmental stages that are critical to tumor development. For effective *in vitro* drug discovery of novel cancer therapeutics, *in vitro* brain tumor models should not only recapitulate tumor biology but culture methods should also be suitable for high-throughput screening (HTS). New technologies and with it the possibilities of more complex screening platforms may be integrated to optimize the model systems for pediatric brain tumors. For example, the recently developed brain cancer-on-a-chip models incorporate multiple tissue types in 3D cultures into microphysiological system (MPS) and provide precise control of a cellular microenvironment and real-time monitoring on cell behavior and response. Nevertheless, while brain cancer-on-a-chip models can better mimic the physiological function of brain, challenges remain. Brain tumors demonstrate profound inter- and intra-tumoral heterogeneity and cellular plasticity to adapt their phenotypes to the surrounding. With more accurate *in vitro* and *in vivo* tumor models, however, it is possible to improve the current low approval rate of anticancer drugs, to offer more treatment options for pediatric brain tumor patients.

Although pediatric brain tumor models have been expanded immensely in the past decades, there is no single model that meets all criteria and thus, experimental design and purpose will need to guide the choice of the brain tumor model (22, 24). The rapid advancement of genomic characterization of pediatric brain tumors and with it new genomic signatures of tumor subgroups add to the complexity of developing precise pediatric brain tumor models. Moreover, in recent years the genome landscape of pediatric brain tumors, both somatic and epigenetic, has been complemented by the analysis of tumor transcriptomes. Despite the plethora of data generated through such approaches, the finding that impaired differentiation of specific neural progenitors is a common mechanism underlying pediatric cancers (185) provides hope that a rational approach towards developing *in vitro* and *in vivo* pediatric brain tumor models can achieve a manageable library of research platforms for the development of impactful therapeutic interventions for pediatric brain cancers.

## Author Contributions

ZL and SL perceived and wrote the manuscript. All authors contributed to the article and approved the submitted version.

## Funding

This work was supported by the Nemours Foundation and the DoBelieve Foundation.

## Conflict of Interest

The authors declare that the research was conducted in the absence of any commercial or financial relationships that could be construed as a potential conflict of interest.
